# History of Newborn Screening for Cystic Fibrosis—The Early Years

**DOI:** 10.3390/ijns6010008

**Published:** 2020-01-31

**Authors:** Georges Travert, Mary Heeley, Anthony Heeley

**Affiliations:** 1 University of Caen Normandy (UNICAEN), 14032 Caen, France; 2 Caen University Hospital, 14040 Caen, France; 3 East Anglian Biochemical Genetic Unit, Peterborough, UK

**Keywords:** newborn screening, immunoreactive trypsin(ogen), dried blood spot, radioimmunoassay, DNA

## Abstract

This review summarises the trajectory of neonatal screening strategies for the detection of cystic fibrosis (CF) using the measurement of Immunoreactive Trypsin (IRT) in dried blood spots (DBS) from 1979 until the beginning of the 21st century when newborn screening (NBS) programmes started to spread throughout many countries, using IRT measurement combined with a CF genotype analysis of DBS.

## 1. The Background

In the 1950s, Dr. Harry Shwachman, paediatrician at Boston Children’s Hospital, recognised that early diagnosis was an important factor underpinning the optimal outcome for cystic fibrosis (CF) patients, receiving both nutritional support and an aggressive treatment of lung infections [1]. However, at this time, no neonatal screening test had been described.

The first attempts at newborn screening (NBS) for CF were performed in the 1970s [2] and were based on a semi quantitative measurement of the albumin content in meconium (BM test). However, elevated meconium albumin levels are a consequence of exocrine pancreatic insufficiency, and pancreatic sufficient CF neonates could not be detected (false negatives). The test also had a very high false positive rate, especially among preterm newborns. Due to its lack of specificity and sensitivity, screening trials with this test were not widely implemented; the exception being where the meconium specimens could be delivered directly from the maternity ward to a laboratory, usually closely associated with a CF clinical centre, where more elaborate testing could take place [3].

The detection of faecal tryptic activity using artificial peptide substrates was next in line for promotion as a potential screening test [4]. Although this eliminated some of the problems associated with meconium testing, the inability to detect pancreatic sufficient neonates would remain a problem. However, two contemporary developments brought these lines of investigation to an abrupt end, as described in the next section. 

These and other issues of the period have been reviewed in detail elsewhere [5].

## 2. New Neonatal Screening Strategies Emerge

Newborn population screening for inherited/congenital diseases such as phenylketonuria (PKU) and congenital hypothyroidism (CHT) had been widely implemented by the late 1970s. Biochemical screening on this scale had been enabled through the innovative work of Robert Guthrie [6], who demonstrated that phenylalanine could be measured accurately in minute amounts of blood that were obtained by heel-prick, collected, and dried on absorbent paper (DBS). Dried blood had the advantage of conferring good stability of the analyte during transport to the laboratory and for later storage. Moreover, these specimens were shown to be suitable for the measurements of analytes in the nanogramme range, such as hormones, using the relatively new techniques of radio-immunoassay (RIA) as Jean Dussault in Quebec, Canada, demonstrated in 1975 with a NBS test for CHT [7].

The 1970s also saw a surge of interest in the role of exocrine proteins in gastrointestinal physiology and pathology. RIA based methods were developed, which were sufficiently sensitive to detect the extremely low concentrations present in the circulation. The pancreatic zymogen trypsinogen was one of these proteins, and the immediate clinical interest in this assay stemmed from its potential for use in the differential diagnosis of pancreatic disease. With this objective in mind, a number of commercial diagnostic companies had, by the late 1970s, developed RIA reagents for serum trypsin(ogen) (IRT) measurement. Although older CF patients with overt pancreatic insufficiency had subnormal serum IRT levels, surprisingly, IRT levels in early infancy were elevated irrespective of the patient’s pancreatic functional status.

The collection of liquid blood and the separation of serum was a cumbersome procedure for neonatal biochemical screening purposes. Would the use of DBS, of proven reliability in other established NBS protocols, also be suitable for the measurement of IRT? The answer to this question came in 1979 from the laboratory of the Department of Paediatrics at Auckland (NZ) Medical School.

The short report by Crossley and co-workers [8] was notable not only for their development of an assay of sufficient sensitivity to measure IRT in dried blood spots but also that IRT was sufficiently stable in DBS form for it to be measurable after storage for many months or even several years. In this study, DBS IRT levels were able to clearly distinguish each of 23 CF neonates from two controls randomly selected in the same batch of Guthrie cards, despite the cards having been stored at room temperature for up to seven years. Therefore the repositories of DBS cards which had been used for PKU/CHT screening would be a valuable resource for the retrospective testing of newborn DBS of infants whose later diagnosis of CF had been established solely on clinical grounds, and in whom the clinical history would be well documented.

The importance of this seminal paper and the almost instantaneous confirmation of its findings in several laboratories cannot be overestimated. The retrospective DBS testing of historical CF infants clearly demonstrated that pre-symptomatic detection of the condition was possible. Even so, it remained unclear how efficient the DBS IRT assay would be in the prospective NBS setting. Additionally, there were aspects of the assay described by Crossley which were unsuitable for routine newborn population screening purposes, i.e., the size of blood spot required. This problem was quickly overcome [9], and the scene was set for prospective screening trials to begin.

## 3. The Two-Stage IRT Prospective Screening Trials (IRT–IRT) 1979–1989

In 1980, apart from the ongoing work in Auckland, there were two European screening laboratories that had the necessary technical and clinical infrastructure in place to incorporate IRT screening alongside their established PKU/CHT programmes. These laboratories were at Caen (France) and at Peterborough (UK), responsible for screening the newborn population of Normandy and East Anglia respectively. At the time, there were two commercially available serum IRT assays, both of which had been independently adapted for DBS IRT screening in these French and UK laboratories (Hoechst Behring Germany in Caen and Sorin Biomedica Italy in Peterborough).

For various complex technical reasons, particularly the lack of an internationally accepted standard preparation of human trypsin(ogen) the results obtained by these different RIAs would not be directly comparable. Each laboratory had to determine, for its own newborn population, the DBS IRT concentration that would provide an optimal screening cut-off.

Moreover, it appeared that hypertrypsinaemia occurs frequently in non-CF neonates during the first days of life, declining rapidly thereafter, whereas the hypertrypsinaemia of CF persists to some degree for several months. Thus, a few infants would have to be re-tested, preferably within the following 1–2 weeks, and, again, an optimal screening cut-off would have to be established for these older infants; those infants with a DBS IRT level above this cut-off level would be referred for diagnostic sweat testing and clinical assessment. Carrying out a sweat test on 4–6 week-old infants by the standard Gibson–Cooke procedure is difficult, cumbersome, and time consuming. In the early 1980s, an innovative sweat collection system was developed commercially, which greatly facilitated the testing of small infants in screening trials [10]. 

It was gratifying to find, as early as 1980, that the results obtained from the trials in Normandy and East Anglia with different assays were producing similar results, in particular, acceptable sensitivity and specificity for the detection of CF infants with low retest rates [11,12]. These preliminary results had, quite independently, in 1980 been disseminated to audiences of paediatricians and clinical biochemists known to be interested in this field of investigation (at Caen in October and London in November). Together with the ongoing work in Auckland (NZ), this led to a burgeoning of two-stage IRT screening trials in other countries, and, as a result, data began to accumulate more rapidly, particularly from those laboratories whose screening hinterland was more populous. Among the latter were New South Wales, Australia, Colorado, USA, and Alto Adige/Veneto, Italy. Initiating these trials, respectively, were Bridget Wilken (Sydney), Keith Hammond (Denver), and Gianni Mastella (Verona).

Two other trials with different objectives, namely to determine whether NBS was clinically effective, began in the mid-1980s. One of these, carried out in Wales/the West Midlands region (UK), elected to screen a large neonatal population for CF using the two-stage IRT method but only on alternate weeks. The other, undertaken in Wisconsin (USA), was an ambitious randomised control trial (RCT) in which half the results of the initial IRT screening test were randomly and anonymously blinded for a period of four years. In the active group, infants with positive IRT singleton test results were immediately referred for sweat testing and, if appropriate, clinical follow-up. However, these trials contributed little useful information regarding the efficacy of the IRT–IRT protocol because, in the case of the former, IRT testing was delayed for 3 weeks after the blood had been drawn, and because of the latter’s aforementioned design [13,14].

As work progressed, it became necessary to convene meetings that would allow investigators to compare results in a timely manner. The first international round table discussions occurred in Peterborough in 1987, but, unfortunately, the sponsorship was insufficient to bring colleagues from the Antipodes. A more generous sponsorship, probably combined with the certain prospect of better food in Normandy, resulted in a widely attended conference with exceptionally fruitful discussions in Caen 1988. The issues addressed at these meetings were as follows: (1) IRT assay methodology. (2) The early nutritional status and respiratory function of the screened cohorts. (3) Optimal shared care between regional CF clinical centres and local paediatricians. 

At that time, 9 laboratories from 7 countries had each screened in excess of 100,000 newborns, and although a majority of these had consistently achieved satisfactory test specificity and sensitivity, others had not. (The data were collected personally by G. Travert and reported in the proceedings of the International Conference: Mucoviscidose, Dépistage néonatal et Prise en charge précoce. Travert G (ed) Université de Caen 1988). The predictive value of a positive (IRT–IRT) test result ranged from 25%–86%, and a retest rate of the initially screened population varying between 0.3%–4.7%.

The reasons for these discrepancies could not be attributed to the type of RIA employed; nevertheless, these assays were inherently prone to sporadic technical error. Other likely confounding variables were the age of the initial and recall testing, age-related screening cut-offs, and the quality of DBS provided for screening, including the very high risk of contamination. 

These and other issues relating to DBS IRT screening have been reviewed in more detail elsewhere [15,16]. Whatever the reasons for the variable results, the need for a within- and between-laboratory performance indicator had been unanimously advocated at a meeting convened in 1985 by G. Mastella in Verona, the organization of which was entrusted to the laboratories of Caen and Peterborough.

The IRT International Quality Assurance Scheme (IRTIQAS) began in 1987 with 16 laboratories from 6 countries. Dried blood spots were prepared from the blood of pancreatitis patients for elevated levels, and often laboratory staff for the control levels, and were distributed monthly. Because different reagents, techniques, and variations of the trypsin antigen were being used by the 16 participants, absolute values could not be compared. However, the scheme gave an indication of within- and between-assay performance and an assurance that laboratories had chosen the appropriate cut-off to distinguish a CF neonate with a minimum proportion of false positives. By 1990 there were 40 laboratories from 8 countries, a clear indication that the scheme was beneficial to laboratories in determining whether their CV and bias were consistent, at which point the manufacturer’s agreed to contribute to the running costs. IRTIQAS was not ideal because the utilized DBS could not be obtained from CF newborns because the volume of blood needed was prohibitive. The lack of an international reference standard was a major drawback. The preliminary results emanating from this scheme were presented at the 1988 Caen Conference and in more detail at the later (1990) International Conference organized by K. Hammond at Estes Park, Colorado [17].

In order to eliminate the multistep, error prone, manual process and radiochemical facilities required for RIA, alternative immunoassay technologies were being introduced in diagnostic clinical chemistry. One of these utilised solid phase monoclonal antibodies, a second chemically labelled antibody and an enzyme linked signal amplification system. Assays of this type could be carried out in multi well antibody-coated microtitre plates with much enhanced and simplified sample throughput. Biochemists at the Queensland (Australia) neonatal screening laboratory in Brisbane had developed such an assay for DBS IRT [18] and a commercial version was launched at the 1988 Caen meeting. The latter generated much interest and some CF screening laboratories changed to this methodology abruptly, causing further confusion in the quest to determine which screening modality was most efficacious in the long term.

In the concluding address of this conference in Caen, the eminent geneticist Jean Frezal predicted that in the future genetic analysis would underpin neonatal screening for CF; a prescient prediction, because, within twelve months, the *CFTR* gene and its main mutation F508del had been described [19]. New horizons for newborn CF screening had been opened up.

## 4. IRT-DNA from 1990

Polymorphic alleles closely associated with the *CFTR* gene had been studied as a potential adjunct to IRT screening in the Normandy neonatal population with some success [20]. However, it was the elucidation of the *CFTR* gene structure and the identification of the F508del mutation with high prevalence in the CF population that provided the stimulus for virtually every major screening centre to embrace molecular genetic analytical techniques. Would the introduction of DNA analysis into the IRT–IRT protocol improve the screening test performance? Would it enable the recall second IRT test to be abolished? Results from some preliminary work were presented at the 1990 International meeting in Colorado. Somewhat surprisingly, the Adelaide laboratory of the South Australian Regional Programme, reported that they had already implemented IRT-DNA screening [21]. They had used a low cut-off (99th centile) on 5-day old infants followed by DBS DNA analysis for the mutations F508del and I507del, i.e., on 1% of the neonatal population. Whilst this protocol eliminated the recall of infants for further IRT testing, it could be cogently argued that it posed a number of problems: (a) sensitivity was limited to 94% (gene prevalence related), (b) all heterozygous infants had to be sweat tested and the parents of those infants with normal results (86%) were offered genetic counselling, (c) those infants that screened IRT positive, in whom either of these mutations was absent (94%), were reported “CF not indicated”; rather tenuously in view of the imposed limit of sensitivity. Most centres running IRT–IRT protocols had, by this time, achieved better sensitivity, fewer infants were recalled for sweat testing, and none of the dilemmas associated with the detection of heterozygotes arose.

At the same meeting, the Caen laboratory group reported investigations, both retrospective and prospective, with IRT-DNA (F508del) and their results showed convincingly that the introduction of DNA analysis, with its present limitations, would not be beneficial as an adjunct to IRT screening if the primary purpose was to maximise the detection of CF infants as soon as possible after birth. Nevertheless, the South Australian work was important in demonstrating the feasibility of IRT-DNA neonatal screening in a routine setting. Its effectiveness would undoubtedly be improved with the inclusion of more CF mutations in the IRT-DNA protocol or in the inclusion of a third stage IRT-DNA-IRT [22]. 

An equally important contemporary finding from the Caen laboratory resulted from their investigation of F508del in the DBS of infants who had screened IRT negative but whose DBS IRT levels approached that of the designated discriminatory cut-off, i.e., the 99.5^th^ centile. In the general French population, the prevalence of F508del healthy carriers is 2.5%–3%. Amongst neonates with low IRT levels, it was 0.5% but progressively reached 10–11% in healthy newborns with IRT levels just below or just above the cut-off [23,24]. The practical consequence of these findings was that any trend to lower the IRT cut-off value, in order to improve the screening sensitivity, would inevitably result in an increased detection rate of healthy carriers. Therefore, as early as 1990, potential problems associated with IRT-DNA screening had emerged. It would take another decade of more adjustments to decide how many of the increasing number of known *CFTR* gene mutations should be included in the screening process.

Unsurprisingly, many of these problems remain unresolved, including the category of screened patients now called CFSPID (CF Screen Positive, Inconclusive Diagnosis) in Europe and CFTR-related Metabolic Syndrome (CRMS) in the USA.

## 5. The Tide Turns 1998

At the instigation of the directors of the Normandy and New South Wales screening laboratories, a further meeting was convened in 1998 at Caen to mark the approaching second decade of newborn screening for CF and to take stock of progress over this period. 

From their inception these International conferences had included the work of clinical psychologists, nutritionists, paediatricians, and CF nurses who had been involved with the individual trials [25,26]. Much had been learned about managing the social and ethical issues of CF screening, of the early natural history of the disease and its clinical management, all of which is covered in Jim Littlewood’s comprehensive on-line historic review of CF [27].

Throughout the 1990s, more robust screening strategies had emerged by increasing the number of CFTR mutations in the DNA analysis. The DBS IRT assay had been substantially improved by the Wallac Co. in Finland, using more selective monoclonal antibodies and an extremely sensitive labelling technology with an analytical system that was already widely used in neonatal screening laboratories for the detection of CHT.

On balance, it seemed that the work that began in the early 1980s had been successfully concluded. The way forward would, in part, depend on local population demographics, ethical considerations, and the ability of health services to deliver optimal treatment for the condition. All that remained to further the case for CF newborn screening was incontrovertible evidence that early diagnosis and clinical intervention conferred significant future health benefits for these infants. That evidence was provided in the long-awaited results of the meticulous RCT conducted by Philip M. Farrell and colleagues in Wisconsin. While presenting these results in Caen in 1998, he concluded that “the burden of proof is now on those who argue against neonatal screening for CF” [26] (p.250).

This gauntlet was unlikely to be taken up by any serious contenders; the results of the Wisconsin trial were confirmation of what a whole generation of paediatricians caring for these patients already knew instinctively. It was also unlikely that any government public health service agency would ignore the evidence. Several NBS laboratories, as early as 1988, had secured health service funding from their respective state/regional public health authorities for the inclusion of CF in their NBS programme, which was justified by the short term clinical and diagnostic cost benefits gained. Before commissioning new public health screening services, national government agencies would normally seek stakeholder consensus for the best practice guidelines; the inclusion of molecular genetic analysis in the current CF NBS protocol presented a novel and more complex issue than had hitherto arisen in NBS commissioning. Unsurprisingly, the time taken to introduce CF NBS at a national level varied considerably, for example, in France by 2003 and four years later in the UK, but by the end of the decade all affluent countries had introduced CF NBS. 

## 6. Conclusions

This historical review focuses on the crucial stages in the development of a reliable neonatal screening test for the early detection of cystic fibrosis in the pre-symptomatic stages of this debilitating disease. Initiating this endeavour was the conviction of many paediatricians with expertise in the treatment of these patients, as the earlier a diagnosis was made, the better the clinical outcome. It seemed particularly fitting that 20 years of work undertaken to justify CF NBS should come to fruition at the beginning of a new millennium and that it should coincide with recent advances in CFTR molecular genetics. The better understanding of CFTR structural/functional relationships would undoubtedly pave the way for new and more effective treatments for CF children born in the 21st century, in addition to the benefits that NBS would provide. The generation of paediatricians and clinical biochemists who had pioneered this work internationally had reason to be satisfied with the outcome of their efforts. CF NBS had at last come of age. The inclusion of DNA in the post 1990 CF NBS protocols, was primarily to overcome a problem inherent in IRT–IRT, i.e., the need to retest about 1 in 250 infants at 2–3 weeks of age. Unanswered was the question—could IRT-DNA in the longer term present its own problems? Perhaps a review of CF NBS in the following decades will reveal the answer.
